# Intestinal Uptake and Tolerance to Food Antigens

**DOI:** 10.3389/fimmu.2022.906122

**Published:** 2022-06-10

**Authors:** Yuhong Xiong, Guifeng Xu, Mingwu Chen, Hongdi Ma

**Affiliations:** ^1^ Department of Pediatrics, The First Affiliated Hospital of University of Science and Technology of China (USTC), Division of Life Sciences and Medicine, University of Science and Technology of China, Hefei, China; ^2^ Institute of Immunology, The Chinese Academy of Sciences (CAS) Key Laboratory of Innate Immunity and Chronic Disease, School of Basic Medical Sciences, Division of Life Sciences and Medicine, University of Science and Technology of China, Hefei, China

**Keywords:** food antigens, food allergy, paracellular pathway, transcellular pathway, intestinal barrier, oral tolerance, intestinal immune system, gut microbiota

## Abstract

Food allergy is a growing concern due to its increasing world-wide incidence. Strict avoidance of allergens is a passive treatment strategy. Since the mechanisms responsible for the occurrence and development of food allergy have not yet been fully elucidated, effective individualized treatment options are lacking. In this review, we summarize the pathways through which food antigens enter the intestine and review the proposed mechanisms describing how the intestine acquires and tolerates food antigens. When oral tolerance is not established, food allergy occurs. In addition, we also discuss the contribution of commensal bacteria of the gut in shaping tolerance to food antigens in the intestinal tract. Finally, we propose that elucidating the mechanisms of intestinal uptake and tolerance of food antigens will provide additional clues for potential treatment options for food allergy.

## Introduction

Globally, there is an increasing incidence of food allergy that affects the quality of life of those affected (1–3). Food allergies have been identified at all ages. Food allergy affects approximately 6% of children and 3 to 4% of adults (4). Allergies to certain foods that start in childhood can persist into adulthood, and new allergies can occur at any stage of life (5). For example, 40 to 60% of fish or shellfish allergies begin in adulthood (6). Peanut allergy affects nearly 5 million adults in the United States, and about one in six individuals with peanut allergy experience their first episode in adulthood (7).

Although food allergy has a wide incidence and is considered a substantial public health burden, its diagnosis and treatment are still inadequate due to the limitations of research into its pathogenesis (8) and the understanding of the mechanisms triggering food allergies (9). First, there are no clear and uniform diagnostic criteria. In addition, most symptoms of food allergy are not typical, such as cough, diarrhea, abdominal pain, and vomiting, which are similar to the symptoms of many other clinical diseases and are often ignored or misdiagnosed (10). Second, because of the lack of understanding of the pathogenic mechanisms of food allergy, there is a lack of safe and effective treatment options for individuals diagnosed with food allergy. The only safe and effective method for patients with a food allergy diagnosis is to strictly avoid the allergic antigens (11, 12). However, there is widespread concern that a strict and limited diet in patients with food allergies can lead to nutritional deficiencies and growth failure in children (13). Recently, allergen-specific immunotherapy has also emerged, and involves the administration of allergic antigens orally, sublingually, or epicutaneously to induce immune tolerance to allergens. Although allergen-specific immunotherapy has made considerable progress in the treatment of food allergies, during the course of clinical treatment, allergen-specific immunotherapy has obvious limitations in efficacy, safety, and durability (14). Thus, an in-depth exploration of the pathogenic mechanism of food allergy is necessary to further improve and optimize treatment plans.

It is well-known that the primary role of the intestinal mucosa is to act as a barrier to prevent harmful substances from entering the digestive system. Furthermore, as a selective filter, the intestinal mucosa allows the necessary dietary nutrients, water, and electrolytes to be diverted from the lumen into the blood circulation of the intestine (15). Although considered as foreign antigens, food antigens are selectively filtered into the circulation by the intestinal mucosa without triggering a defense immune attack response in the intestines but induces immune tolerance. Nevertheless, the disruption of this tolerance mechanism will lead to food allergies. Herein, we focus on how food antigens pass through the intestinal barrier and how they are acquired and tolerated by the intestinal immune system. We also discuss situations in which food allergy occurs when the intestinal immune regulation is disturbed. Finally, by summarizing and discussing these studies on food allergy, we hope to provide more clues to stimulate fundamental research and clinical applications in the field of food allergy.

## How Do Food Antigens Cross the Intestinal Barrier?

Structurally, the intestinal barrier can be divided into three layers. The outer layer is the mucus layer that is symbiotic with intestinal microorganisms, the central layer is a specialized single cell layer consisting of epithelial cells, and the inner layer is the lamina propria (LP) composed of innate and adoptive immune cells. The mucus layer has a Sieve-like structure, among which mucins secreted by goblet/mucinous cells cover the intestinal epithelium (16). Mucins secreted in the mucus layer can protect intestinal epithelial cells from digestive enzymes and function as a defense barrier to prevent the invasion of foreign microorganisms (17–19). The central layer is made up of intestinal epithelial cells that are considered important components of the intestinal defense system and play a key role in the transport of substances into the intestinal tract. A variety of epithelial cell subsets, including absorptive enterocytes, goblet cells, Paneth cells (20, 21), tuft cells (22), enteroendocrine cells (23), microfold cells (M cells), and epithelial stem cells, have unique and specialized characteristics and functions, which cooperatively form a sophisticated epithelial layer against numerous antigens in the lumen (24). A set of highly organized intercellular junction complexes links these intestinal epithelial cells to form intestinal paracellular barriers. These junction complexes are in dynamic balance and are divided into three types: tight junctions (TJs), adherens junctions (AJs), and desmosomes (25). Paracellular barriers composed of these junction complexes function to maintain the integrity of the intestine and also mediate the regulation of nutrients that pass through the intestine through the paracellular pathway. Beyond the mucus layer and the epithelial layer, is the LP, which contains both innate and adaptive immune cells, such as dendritic cells (DCs), macrophages, T cells, and B cells. Immune cells in the LP are involved in both immune defense and immune regulation in the intestinal microenvironment (26). The intestinal barrier composed of these three layers can effectively prevent harmful substances from entering the body. However, this barrier is not completely impenetrable. The intestinal barrier allows foreign nutrients to enter the body to meet growth needs. We will first summarize the pathways through which nutrients from ingested food pass through the intestine.

Food is digested into peptides, amino acids, polysaccharides, monosaccharides, water, electrolytes, and other nutrients through chemical and mechanical activity in the digestive tract. These nutrients, including food antigens, enter the subepithelium through two main pathways, the paracellular pathway and the transcellular pathway.

### Paracellular Pathway

**Figure 1:**
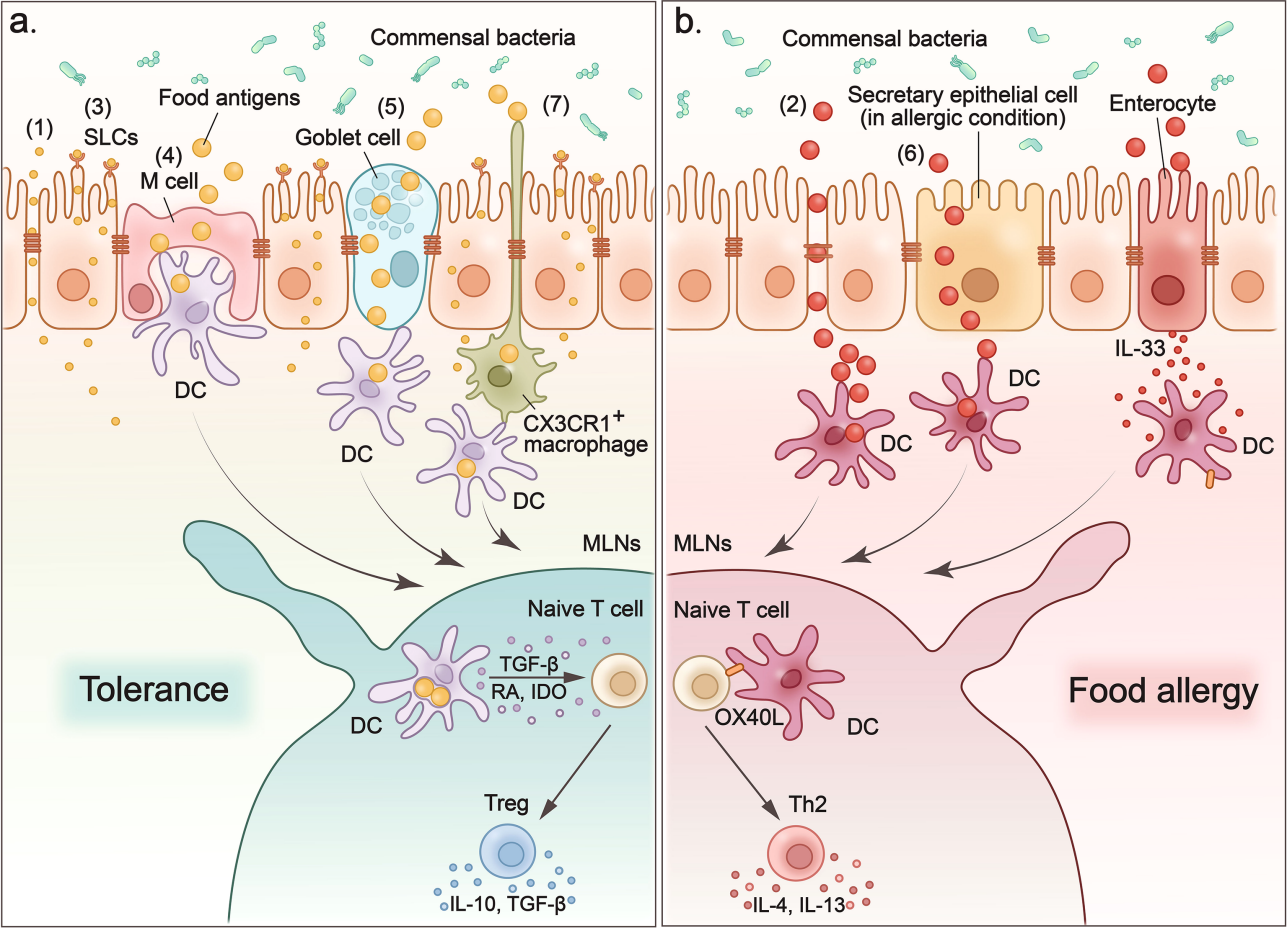
Food antigens cross the intestinal epithelium to induce tolerance or provoke allergy. (1) Small molecules such as electrolytes and water enter the sub-epithelium *via* paracellular pathways. (2) Degradation of tight junctions increases the permeability of the paracellular pathway, which leads to non-selective and uncontrolled entry of macromolecules into the intestinal sub-epithelium and contributes to the sensitization process of food allergies. (3) Small molecules such as amino acids and glucose that are transported across the epithelium by apical brush boundary transporters such as SLCs on enterocytes. (4) M cells sample food antigens, and then present them to dendritic cells. (5) Goblet cells form goblet-cell-associated antigen passage (GAP) to enclose luminal antigens (food antigens and microbial antigens) in internal sack-like vesicles, and then transport them across entire cells to dendritic cells. (6) In food allergy mouse models, secretory epithelial cells function as conduits to allow the transport of food antigens through the epithelium of the small intestine and induce a food-induced anaphylactic reaction. (7) CX3CR1^+^ macrophages rely on CX3CR1 expression to form dendrites to efficiently sample antigens from the intestinal lumen without disrupting the tight junctions between the cells. CX3CR1^+^ macrophages transfer antigens they captured to CD103^+^ DC. **(A)** When food antigens enter the intestinal sub-epithelium through above pathways, they are acquired and processed by dendritic cells. Then DCs migrate to MLNs where they present the processed food antigens and induce tolerance or food allergy. To induce oral tolerance, DCs secrete interleukin-10 (IL-10), transforming growth factor-β (TGF-β), and indoleamine 2,3-dioxygenase (IDO) to induce Tregs and promote their production of IL-10 and TGF-β to maintain tolerance. **(B)** In the case of allergy, oral antigens and adjuvants stimulate the expression of IL-33 in intestinal epithelial cells, which in turn upregulates the expression of the costimulatory molecule-OX40L on DCs. OX40L expression upregulates DCs to promote the TH2 response in the MLN and leads to more severe food allergies.

As mentioned above, the intercellular junction complex located in intestinal epithelial cells is the main mediator that regulates the paracellular pathway. Among the three types of epithelia junction complexes, TJs are composed of transmembrane proteins (27), which interact with each other and with the intestinal immune system, making them the main rate-limiting step in controlling the permeability of the paracellular pathway (28, 29). The TJ proteins, such as zonula occludens (ZO) (30–32), occludins (33), and claudins (34) participate in the formation of TJs and control the permeability of paracellular pathways. Small molecules derived from food nutrients, such as solutes soluble in water, cross the intestinal barrier through paracellular pathways (**Figure 1-(1)**). Paracellular pathways have the selectivity of capacity, charge, and size for the substances they transport. This pathway is highly regulated by TJs to ensure that the transport of materials across the epithelial barrier is strictly controlled. However, when the epithelial barrier is damaged, such as following destruction of TJs, the paracellular pathway becomes nonrestrictive and allows the free passage of ions, water, macromolecules, and even bacteria or viruses. This increases the intestinal permeability and leads to pathological changes (**Figure 1-(2)**). For example, food cysteine proteases degrade the TJ protein occludin, thus increasing the permeability in the paracellular pathway, which can contribute to the sensitization process of food allergies (35). Furthermore, the paracellular pathway is disrupted in patients with inflammatory bowel disease (IBD) due to the reduced expression of TJs and AJs proteins in epithelial cells in the inflammatory zone, such as ZO-1, claudin, and E-cadherin (36). Thus, the paracellular pathway plays an indispensable role in mediating materials into the intestinal subepithelium, and this process is controlled by TJs between epithelial cells. Furthermore, damage to TJs disrupts the paracellular pathway, which leads to increased intestinal permeability and intestinal disorders such as food allergies and IBD.

### Transcellular Pathway

Enterocytes are the main type of intestinal epithelial cells and differ greatly in structure and composition between the small intestine and the colon. In the small intestine, enterocytes have villi that protrude into the lumen. The villi increase the surface area of the intestinal mucosa for better absorption of nutrients. These cells concentrate digestive enzymes (such as pancreatic proteolytic enzymes) on their apical surface, which are involved in the chemical digestion of lipids, carbohydrates and proteins, and absorb these digested nutrients through the apical brush boundary transporters (such as the SLC1A, SLC6A, and SLC7A families) (37). As we mentioned, small molecules such as water (38) and ions (39) can enter the intestinal subepithelium *via* paracellular pathways. They can also enter the intestinal cytoplasm from the apical membrane through epithelial transporters (integral membrane protein pumps or channels) and are discharged from the basolateral membrane (**Figure 1-(3)**). For example, the sodium-dependent transport of glucose (40), alanine (41), and glutamine (42) occurs on the surface of intestinal epithelial cells. However, macromolecules can only enter the cell through vesicles, which are formed by invagination and extrusion of the apical membrane. The vesicle transfer process is called endocytosis (43), which in intestinal cells is limited to pinocytosis (44). A variety of intestinal epithelial cells are involved in these intestinal epithelial-mediated transcellular pathways. Among them, M cells and goblet cells play a pivotal role in internalizing luminal antigens into gut-associated lymphoid tissues (GALTs) and in establishing intestinal tolerance or inducing intestinal immune responses to food antigens. They indiscriminately sample lumen contents, including food antigens, and transport the intact antigens to intestinal DCs to process and present these antigens (37).

M cells are specialized epithelial cells located in the follicle-associated epithelium overlying Peyer’s patches (PP) (45, 46). Their location facilitates M cells to transcytose a wide range of substances, such as food antigens and microbes, to underlying DCs for antigen processing and presentation (47) (**Figure 1-(4)**). In addition, M cells have specialized structures that lack the typical brushlike border and have thinner calyx glycosomes, making it easier to capture large particle antigens and transcytose them by pinocytosis in the fluid phase and by receptor-mediated endocytosis (48). Besides the delivery of intact antigens into the underlying lymphoid tissue of the GALT (49, 50), M cells also participate in Ag processing and presentation based on the observation that GALT M cells express MHC class II molecules and acidic endosomal-lysosomal compartments (51). Suzuki et al. developed an M cell-targeting Ag delivery system by combining antigens OVA with pσ1 protein that is known to bind to M cells in gut and nasal-associated lymphoid tissues (NALT) to investigate the role of M cells in oral tolerance (47, 52, 53). Using this M cell-targeting antigen delivery system, the authors determined that the recombinant protein OVA-pσ1 can induce mucosal unresponsiveness through two main mechanisms: clonal deletion of Ag-specific CD4+ T cells and the induction of acquired type Tregs cells (47). However, additional studies have also reported that oral tolerance could be established even in the absence of PP or the destruction of M cells to facilitate antigen transport to the PP (54, 55). Thus, the role of M cells in the induction of oral tolerance by transporting Ags from the lumen is controversial and further studies of the mechanism are needed.

Goblet cells are specialized mucous epithelial secretory cells. The secretory products of goblet cells, including mucins, trefoil factors, and other proteins, are essential for the integrity of the intestinal barrier and the prevention against the entry of harmful antigens (56). It should be noted that another important role of goblet cells is the formation of a goblet cell-associated antigen passage (GAP) to transport luminal antigens (food antigens and microbial antigens) to antigen-presenting cells (APC) in the LP (57, 58) (**Figure 1-(5)**). Preventing the entry of harmful antigens through mucus secretion and sampling luminal substances into the intestinal immune system through GAP formation are two divergent processes for goblet cells. Molecular mechanisms have revealed that a neurotransmitter called acetylcholine can trigger both mucus release and the GAP process in goblet cells in independent signaling pathways mediated by different receptors (59). This regulation of Ach allows goblet cells to accommodate the dynamically changing demands of the mucosal environment. Goblet cells can also deliver luminal substances, including food antigens and microbial antigens, and induce intestinal tolerance through the GAP process (60). First, goblet cells capture food antigens from the lumen, enclose them in internal sack-like vesicles, and then transport them across the entire cell. Then the APCs in the LP acquire luminal antigens to induce intestinal tolerance by maintaining pre-existing Tregs in the LP, and imprinting tolerogenic properties (60). However, Noah et al. reported that in food allergic mice, secretory epithelial cells, including goblet cells, enteroendocrine cells, and Paneth cells in the small intestine function as conduits to allow the transport of food antigens through the epithelium of the small intestine to the underlying immune cells and induce a food-induced anaphylactic reaction (61) (**Figure 1-(6)**). They also found that these secretory epithelial cell antigen passages (SAP) were induced by the Th2 cytokine-IL-13 in a CD38/cADPR-dependent manner (61). Additionally, blockade of this process reduced the passage of food antigens through the epithelium of the small intestine and alleviated the induction of the food allergic reaction in the intestine (61). Finally, they confirmed that SAP formation driven by IL-13 through the PI3K/CD38/cADPR pathway is conserved in the human intestine, indicating that blockade of this process, such as by inhibiting Th2 cytokines, might represent a potential therapeutic option for food allergy.

## How Are Food Antigens Acquired and Tolerated By The Intestinal Immune System?

When food antigens enter the intestinal sub-epithelium through the above pathways, they are acquired and processed by APCs dispersed in the LP, PPs, and mesenteric lymph nodes (MLN). Oral tolerance to food antigens is often induced in MLNs (55) (**Figure 1A**). When the underlying APCs acquire the food antigens, they process them and present them to immune regulatory cells such as Treg cells in the MLN and induce tolerance (62).

### Food Antigen Capture and Oral Tolerance Induction in the Intestine

As we mentioned above, food antigens can be internalized by M cells or acquired by goblet cell-associated passages. When the food antigens are captured by these specialized intestinal epithelial cells, they will be transferred to the migratory DCs in the intestinal PP (where the M cells deliver antigens to underlying DCs) (63) and LP (where the GAPs transport antigens to DCs) (57).

DCs and macrophages are the two main APCs for food antigens (64, 65). Rescigno et al. observed that DCs can penetrate the monolayers of the intestinal epithelium into the gut lumen by extending the transepithelial dendrites (66). This property provides DCs with access to antigens in the intestinal lumen. CX3CR1^+^ macrophages have also been reported to rely on CX3CR1 expression to form dendrites to efficiently sample antigens from the intestinal lumen (67). These antigen uptake macrophages quickly transfer food antigens to CD103^+^ DCs *via* connexin 43 in the gap junctions (67) (**Figure 1-(7)**).

In general, food antigens are collected directly by intestinal DCs or by CX3CR1^+^ macrophages or other epithelial cells (such as the M cells and goblet cells described earlier) and are delivered to DCs (68).

When loaded with food antigens, DCs migrate to the MLNs where they present the processed food antigens and induce tolerance (**Figure 1A**). Among various subsets of DCs, CD103^+^ DCs have been reported to play an important role in tolerance induction (67). CD103^+^ DCs are derived from circulating monocytes that express the gut homing marker-α4β7 integrin (69). They are located in the LP of the small and large intestine. When they acquire food antigens from the lumen, they migrate to MLNs to induce oral tolerance by activating Tregs. There are several mechanisms through which CD103^+^ DC induce Tregs. For example, intestinal CD103^+^ DCs have been reported to secrete retinoic acid (RA) and transforming growth factor-β (TGF-β) to promote the differentiation of Foxp3+Treg cells (70, 71). CD103^+^ DC also express indoleamine 2,3-dioxygenase (IDO), to sustain and differentiate Tregs, while inhibition of IDO *in vivo* has been reported to reduce Tregs specific to orally administered antigens and to impair the induction of oral tolerance (72).

Besides CD103^+^ DC, resident intestinal macrophages marked with high expression of the CX3C-chemokine receptor 1 (CX3CR1) also help to maintain Foxp3 expression in Tregs in the intestine by secreting IL-10 (73, 74). These intestinal resident macrophages have been proposed to provide additional survival signals for Tregs since they express high level of MHC class II, which enables them to undergo cognate interactions with specific Treg cells (75).

In addition to Treg-mediated oral tolerance, T cell clone anergy and/or deletion are also involved in oral tolerance (76, 77). The modality of oral tolerance induction depends on the dose of food antigens (77). Low dose of antigens induce Treg mediated immune suppression, while high doses of antigens lead to anergy and deletion of antigen-specific T cells (76–78).

### Intestinal Commensal Bacteria Help Establish Oral Tolerance

Intestinal commensal bacteria also provide a large number of non-self-antigens that are tolerated by the intestinal immune system (73, 79, 80). Evidence indicates that the intestinal microbiota is crucial for the development and maturation of the intestinal immune system (81, 82). In particular, intestinal commensal bacteria could help shape intestinal tolerance. In germ-free (GF) mice, the frequency of Tregs and the levels of the anti-inflammatory cytokine IL-10 produced by Tregs are markedly reduced compared to mice free of specific pathogens (83–86). In one study, 17 strains of bacteria from the human gut microbiota were identified as Treg-cell-inducing bacterial strains. Treatment of these 17 Treg cell-inducing strains could alleviate intestinal inflammation, including allergic diarrhea (87). Furthermore, the observation that food allergen sensitization is enhanced in GF mice or mice that have been treated with antibiotics suggests that commensal bacteria are essential for the establishment of oral tolerance (88). Although the mechanisms by which the intestinal microbiota regulate allergic responses to food are not yet fully defined, studies have revealed that the composition of the gut microbiota, metabolites derived from intestinal bacteria and colonization of special functional bacteria are important factors that influence intestinal tolerance to food antigens.

Mechanistically, intestinal bacteria-derived metabolites, including inosine and short-chain fatty acids (SCFAs), are considered key factors promoting Treg differentiation and improve the production of regulatory cytokines such as IL-10 (89–91). In particular, SCFAs, which are produced during the bacterial fermentation of indigestible dietary fiber, have received much attention for their immunoregulatory activity. Butyrate, one of the most abundant SCFAs in the gut, has been reported to induce functional colonic Treg cells through its function to enhance histone H3 acetylation in the promoter and conserved non-coding sequence regions of the Foxp3 locus (92). In addition, it has been reported that SCFAs, particularly acetate and butyrate, could help establish oral tolerance and prevent food allergy by enhancing retinaldehyde dehydrogenase-2 (RALDH2) activity in CD103^+^ DC (93). RALDH2 converts vitamin A to retinoic acid, which promotes the differentiation of naive T cells into Treg cells and contributes to the establishment of oral tolerance (93, 94).

Stefka et al. reported that colonization of a Clostridia-containing microbiota can protect against sensitization to food allergens (88). Colonization of Clostridia induced early production of IL-22 by RORγt+ innate lymphoid cells (ILCs) and T cells in the intestine. This Clostridia-Induced IL-22 reduced the access of food allergen to the circulation (88).

In addition, dysbiosis of the gut microbiota leads to intolerance in the intestine. When the gut microbiota of infants allergic to milk protein was transplanted into GF mice, these recipient mice also showed an allergic response to milk-allergens (95).

## Pathogenesis and Treatment of Food Allergy

The most important role of intestinal immune system is to distinguish innocuous food antigens and commensal microbes from pathogens. They initiate an immune response against pathogens and induce tolerance to food and commensal bacteria. However, a breakdown of the default oral tolerance to food leads to abnormal immune responses and results in food allergy (**Figure 1B**). Many factors including the genetic background, alteration of gut microbiota, food allergenicity and methods of food processing, may trigger a food allergy.

As we mentioned, the intestinal APCs, especially intestinal DCs play a pivotal role in the induction of tolerance. However, stimuli from food components or extrinsic adjuvants could activate DCs to trigger a food allergy. Although the detailed identification of stimuli and their reorganization are not very clear, it has been reported that glycoproteins from the allergenic foods could directly bind to C-type lectin receptors (CLR) on DCs to stimulate immune response to the food allergens (96). For instance, in the peanut induced allergy, the glycoprotein Ara h 1 was identified as the major peanut allergen able to bind to DC specific intercellular adhesion molecule-3-grabbing non-integrin (DC-SIGN), a C-type lectin receptor, on monocyte-derived DCs and subsequently activate DCs to induce allergic immune responses (97). Similarly, hazelnuts, walnuts, and egg whites have also been found to bind to DC-SIGN and related DC-SIGNR to activate DCs and contribute to the development of food allergies (98, 99).

In addition, alarmins such as IL-25, IL-33, and TSLP are also involved in the development of food allergies (100, 101). Among these allergenic alarmins, intestinal epithelial cell-derived IL-33 has been reported to act on different immune cells to expand the allergenic immune response in the intestine. In an allergenic mouse model, oral Ags and adjuvants stimulate the expression of IL-33 in intestinal epithelial cells, which in turn up-regulate the expression of the costimulatory molecule-OX40L in DCs. These DCs expressing upregulated OX40L promote the Th2 response in the MLN and lead to more severe food allergies (102, 103) (**Figure 1B**). In another study, IL-33 secreted by intestinal epithelial cells was found to act on type 2 ILC (ILC2) to enhance their expansion and induce their production of IL-4 (104). The IL-33 signal-stimulated production of IL-4 by ILC2 is indispensable for oral allergic sensitization and anaphylaxis (104, 105). Furthermore, IL-33 acts directly on mast cells to potentiate antigen-driven IgE-dependent degranulation of MC and promotes oral anaphylaxis after epicutaneous sensitization (106).

Based on current understanding of the mechanisms of oral tolerance and food allergy, there have been significant advances in treatment to food allergy, such as allergen specific immunotherapy, vaccines, and non-allergen specific therapies, which provide viable options for patients with food allergies.

For allergen specific immunotherapy, patients with food allergies are treated with their specific allergens to establish the tolerance to these allergens. This process is called desensitization. There are various approaches to treating patients with allergens including oral immunotherapy (OIT), sublingual immunotherapy (SLIT), and epicutaneous immunotherapy (EPIT). Specifically, patients with allergies are treated with their allergens in increasing amounts each time until a maintenance dose is reached, and then this dose is given periodically to patients (107). Compared to other allergen-specific immunotherapies, OIT has a higher efficiency, but also has a higher risk of systemic side effects, which may even require therapeutic intervention. Clinical trials have shown that OIT directed at milk, eggs, peanuts, and wheat allergens is therapeutically effective; however, OIT directed at these allergens generally caused significant adverse effects when the dose is increased (108–110). In addition to concerns about safety, there are many factors that limit the application of allergen-specific immunotherapy. There is a lack of standardization of clinical treatment, including the type of allergen used in the treatment, the administration method, the given dose, and frequency (111, 112). Further research is needed to promote and apply allergen-specific immunotherapy in the clinical treatment of food allergies.

Since the allergenic activity of natural allergen extracts is the most concerning side effect of allergen-specific immunotherapy, and broadly limits its applicability. However, recombinant allergens with genetic modifications that can reduce allergenic activity are produced to improve the safety of the immunotherapies (113). Clinical therapy trials suggest that recombinant allergens are effective for subcutaneous immunotherapy (114–116). Based on the promising results of these clinical trials, the first recombinant allergen-based vaccines will soon be registered and available for routine clinical use in patients with allergies.

Except for allergen-specific immunotherapy, nonallergen-specific therapies for food allergies have been developed, including immune antibody therapy and prebiotics treatment. As an immune antibody therapy, omalizumab has been tested in clinical trials as a monoclonal antibody against immunoglobulin E (IgE) (117). The combination of omalizumab and OIT has achieved promising results for the treatment of food allergies (118). In the future, with an in-depth understanding of the mechanism of food allergy and the identification of therapeutic targets, more targeted antibodies, such as antibodies against Th2 cytokines, will be developed and used in the treatment of food allergy.

Since intestinal microbiosis contributes significantly to allergic states in the intestine, the manipulation of intestinal microbes holds promise for the treatment of food allergy. Preclinical evidence has shown that prebiotics have a positive effect on remission of food allergy. For example, dietary supplementation with fructo-oligosaccharides, an immunomodulatory prebiotic, significantly improved allergic intestinal inflammation in OVA23-3 TCR-transgenic mice fed with an OVA-containing diet (119). Supplements consisting of *Lactobacillus paracasei* L9 reduced allergic responses in mice allergic to β-lactoglobulin (120). Oral administration of *Lactobacillus murinus* restored the deterioration of the intestinal flora in food-allergic mice and alleviated allergic reactions (121). Although prebiotic and probiotic trials are promising in food allergy treatment, there is currently no solid evidence to support the preventive or therapeutic effects of prebiotics and probiotics in relation to clinical food allergies. Therefore, future studies should uncover more specific details and mechanisms for the treatment of food allergies, while optimal functional probiotic strains should be selected and isolated for this approach.

## Conclusion

The incidence of food allergy worldwide has increased progressively. When food allergies are diagnosed, there are limited treatment options for patients. Strictly avoiding allergens is one of the few safe and effective treatments in clinical application. However, such treatment is considered as a passive option with significant shortcomings. The limitations of clinical treatments for food allergies are largely attributable to unclear disease mechanisms. In recent years, significant progress has been made in elucidating the mechanisms involved in food antigen uptake and oral tolerance induction in the intestine. In this review, we summarized the pathways in which food antigens cross the intestinal epithelium and the processes through which they are transferred to the sub-epithelial compartment to induce tolerance or to provoke allergic reactions in the intestine. However, more mechanistic details need to be explored regarding processes associated with the promotion of clinical manifestations associated with food antigen-triggered anaphylaxis. More importantly, promising clinical strategies have been proposed, such as allergen-specific immunotherapy, vaccines, and non-allergen-specific therapies, which may provide additional viable options for patients with food allergies.

## Author Contributions

HM and YX wrote the manuscript. GX provided important advice and suggestions. HM and MC supervised the writing. All authors contributed to the article and approved the submitted version.

## Funding

This work was supported by the National Natural Science Foundation of China (#82171783, # 81871284).

## Conflict of Interest

The authors declare that the research was conducted in the absence of any commercial or financial relationships that could be construed as a potential conflict of interest.

## Publisher’s Note

All claims expressed in this article are solely those of the authors and do not necessarily represent those of their affiliated organizations, or those of the publisher, the editors and the reviewers. Any product that may be evaluated in this article, or claim that may be made by its manufacturer, is not guaranteed or endorsed by the publisher.
